# Unique Crystal Structure of a Self-Assembled Dinuclear Cu Peptoid Reveals an Unusually Long Cu···Cu Distance

**DOI:** 10.1021/acsomega.4c06987

**Published:** 2024-09-26

**Authors:** Guilin Ruan, Natalia Fridman, Galia Maayan

**Affiliations:** Schulich Faculty of Chemistry, Technion−Israel Institute of Technology, Haifa 32000, Israel

## Abstract

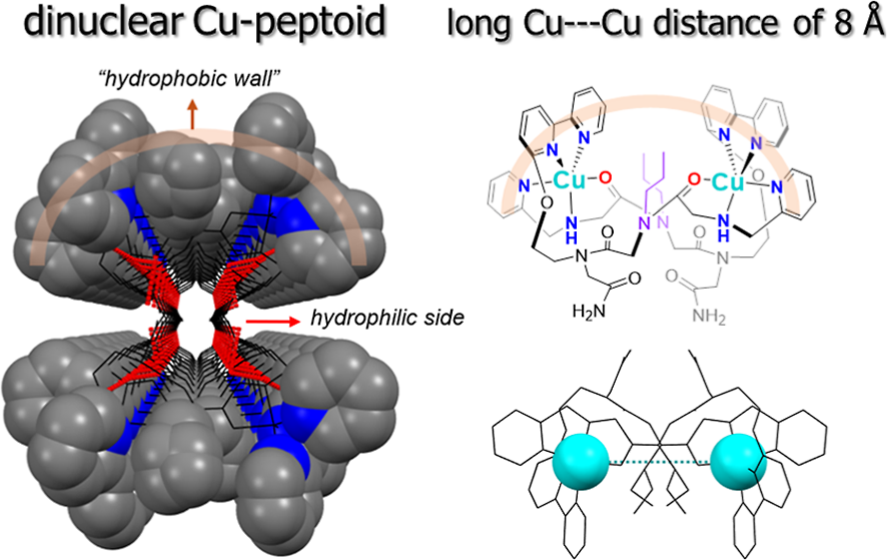

Studies on a series of molecular dicopper peptoid complexes showed that the Cu···Cu distances measured in X-ray single-crystal diffraction are typically in the range of 4.2–6.9 Å. Herein, we designed a new peptoid, **L1**, having 2,2′-bipyridine, propyl, and pyridyl side chains and discovered that although it forms a typical dicopper self-assembled structure (complex **1**), the Cu···Cu distance is exceedingly long −8.043 Å. By analyzing its structure and surface properties in comparison to a control Cu-peptoid complex (**2**), in which the pyridyl side chain is modified by an ethanolic side chain, we suggest that the long Cu···Cu distance is contributed by the hydrophilic–hydrophobic interaction influenced by the pyridyl side chain and the steric hindrance of the propyl side chain. This result may motivate the use of dinuclear Cu peptoid complexes for wider applications, such as cooperative catalysis and luminescence.

## 1 Introduction

Peptoids—N-substituted glycine oligomers—are versatile scaffolds that are synthesized from primary amines rather than from amino acids.^1^ Thus, numerous functional groups can be efficiently incorporated within peptoid scaffolds, enabling the design of various structures.^2−4^ Among the different functional groups are metal binding ligands, which upon metal coordination lead to the formation of metallopeptoids.^5−7^ Specifically, Cu-peptoids demonstrated high potential in diverse applications such as nanotransport materials,^8^ catalysis,^9−11^ electrocatalysis,^12−15^ Alzheimer disease therapeutics,^16,17^ and drug delivery.^18^ The versatility of Cu-peptoids arises from their tunable structures, which are mediated by their peptoid ligands.^19^ For example, polypyridyl and alcoholic scaffolds have been successfully incorporated within peptoids, resulting in the formation of macrocyclic dinuclear Cu-peptoids.^13,19,20^ Typically, these macrocyclic structures self-assemble with two Cu ions and two peptoid ligands in a short time and exhibit high thermodynamic stability in organic/aqueous solutions. According to X-ray single-crystal structures, the Cu···Cu distance in dinuclear Cu-peptoids is in the range of 4.2–6.9 Å, depending on the sequential side chains of peptoids.^8,13,19,20^ The range of Cu···Cu distance within self-assembled (nonpeptoidic) structures has been shown much broader, offering potential for selective catalysis,^21−23^ host–guest interactions,^24,25^ luminescence,^26−28^ and more.^29,30^ We therefore believe that a wider range of metal–metal distances within metallopeptoids will enhance their utility.

**Figure 1:**
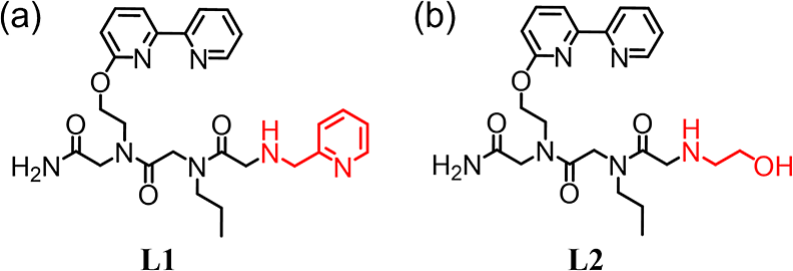
Molecular structures of the designed peptoid ligands **L1** (a) and **L2** (b).

In this study, we report on a dinuclear Cu-peptoid, compound **1**, assembled from the peptoid **L1**; this Cu-peptoid displays a Cu···Cu distance of 8.043 Å, as seen from X-ray analysis of its single-crystal structure, which is much larger than the previously reported Cu···Cu distances within metallopeptoids.^8,13,19,20^**L1** consists of 2,2′-bipyridine, propyl, and pyridyl side chains at the C-terminus, middle, and N-terminal positions, respectively (see Figure 1a). A control Cu-peptoid, compound **2**, having an ethanolic group instead of a pyridyl side chain at the N-terminal (see Figure 1b) position was also prepared; the Cu···Cu distance in **2**, as determined by the same X-ray analysis like **1**, was much shorter—only 4.550 Å.^13^ This result indicates that a single modification at one position along the peptoid scaffold leads to a large difference of 3.493 Å between the Cu···Cu distances found in the crystal structures of **1** and **2**. Further analysis of these crystal structures reveals that the hydrophilic–hydrophobic interaction orients the hydrophobic propyl side chain to different directions in each complex.^31,32^ In **1**, this orientation creates steric hindrance between the Cu ions, increasing the distance between the two Cu ions. In contrast, in **2**, the propyl side chain faces outward from the macrostructure, enabling a water molecule to interact with the two Cu centers, acting as a bridge between the two Cu ions in a short distance.^13^ These results indicate the significant impact that subtle changes in the peptoid sequence can have on the structural properties of di-Cu peptoids.^33^

## 2 Experimental Methods

### 2.1 Materials and Methods

Rink amide resin was purchased from Novabiochem; ethanolamine and 6-bromo-2,2′-bipyridine were purchased from Acros organics, Israel; *N*,*N*′-diisopropylcarbodiimide (DIC) and bromoacetic acid were purchased from Sigma-Aldrich. The other chemicals used in this work were purchased from commercial sources and used without additional purification. Synthesis of 2-(2,2′-bipyridine-6-yloxy) ethylamine and ethanolamine protection were prepared according to the literature.^20^ The solvents used were high-performance liquid chromatography (HPLC) grade. High-purity deionized water was obtained by passing distilled water through a nanopore Milli-Q water purification system. Peptoid ligands were analyzed by reversed-phase HPLC (analytical C18 column, 5 μm, 100 Å, 2.0 × 50 mm) on a Jasco UV-2075 instrument. A linear gradient of 5–95% ACN in water (0.1% TFA) over 10 min was used at a flow rate of 0.7 mL/min. Preparative HPLC was performed using a Phenomenex C18 column (15 μm, 100 Å, 21.20 mm × 100 mm) on a Jasco UV-2075 instrument. Peaks were eluted with a linear gradient of 5–95% ACN in water (0.1% TFA) over 60 min at a flow rate of 5 mL/min. Mass spectrometry for peptoids was performed on a Waters LCT Premier mass and Advion expression mass under electrospray ionization (ESI).

### 2.2 Preparation of Peptoid Ligands **L1–L2**

The peptoids **L1** and **L2** were prepared using the submonomer solid-phase synthesis.^34^ Two-step reactions, acylation and nucleophilic attack substitution, were repeated iteratively to obtain the desired peptoid oligomers. All oligomers were synthesized at room temperature. Initially, 100 mg of Rink amide resin (Novabiochem; 0.81 mmol/g) was set to swell in dry dichloromethane (DCM, 4 mL) for 40 min and then washed three times with anhydrous DMF (3× 1 mL). Then, the deprotection of resin was performed by the addition of a 20% piperidine solution (2 mL) and shaken for 20 min. Following the reaction, piperidine was washed from the resin using DMF (3× 1 mL). The first acylation was initiated by adding 20 equiv of bromoacetic acid (1.0 M in DMF) and 24 equiv of DIC and shaken for 20 min. Subsequently, the reagents were washed by DMF (3× 1 mL), and 20 equiv of desired primary amine (1.0 M in DMF) was added for stepwise nucleophilic attack substitution. Later, the excess amine after the reaction was washed by DMF (3× 1 mL). This two-step reaction cycle was repeated, until the desired sequence was completed. It is worth noting that the reaction times of the amine displacement were modified as follows: 2-(2,2′-bipyridine-6-yloxy)ethylamine was shaken particularly for 5 h.^20,35^ After that, the resin was cleaved from the solid support by using 95% TFA in water. Finally, the cleavage cocktail was purified by HPLC (>95% purity). The collected solution from HPLC was then freeze-dried at −35 °C in a lyophilizer overnight. Finally, off-white transparent solid or purely white powder was obtained. The product form depends on its hygroscopicity. The molecular weight and the purity of the solid product were determined by electrospray mass spectrometry (ESI-MS) and analytical HPLC, respectively. The details of this data for **L1** are shown in the Supporting Information. Peptoids **L2** has been previously reported by our group.^13^

### 2.3 Complexation and Crystallization of **1**–**2**

Each of the purified peptoid oligomers (**L1**, or **L2**) was dissolved in *n*-propanol (ca. 0.05 mmol of peptoid per mL of solvent), and the solution was stirred for 10 min. Later, 1 equiv of Cu(ClO_4_)_2_·6H_2_O was then added into the solution and stirred for 2 h in ambient condition. Greenish-blue precipitate was obtained and isolated from the solution by centrifugation. The isolated solid was washed by *n*-propanol (1 mL) at least three times until no color shows up in the filtrate. Finally, the washed solid was redissolved in acetonitrile/water (the complex was dissolved in 0.5 mL of acetonitrile, and a few drops of water were added until a clear solution was obtained) and crystallized with the slow evaporation of the solvent. The crystals were then analyzed by single-crystal X-ray diffraction, and the molecular weight was determined by ESI-MS in acetonitrile/water mixture. The details of the data for **1** are shown in the Supporting Information, while **2** has been previously reported by our group.^13^ CCDC 2351756 and 2084880 contain the supplementary crystallographic data for this paper. This data can be obtained free of charge from the Cambridge Crystallographic Data Center.

### 2.4 Single-Crystal X-ray Diffraction

Low temperature (100 or 200 K) diffraction data were collected by using a Bruker APEX-II diffractometer coupled to an APEX II CCD detector with graphite monochromatic Mo Kα radiation (λ = 0.71073 Å) and a cryostat system equipped with an N_2_ generator. The crystals were removed from the solution, attached to a loop of nylon fiber with an antifreeze reagent (paraton-N, Hampton research), and mounted onto a goniometer. All diffractometer manipulations, including data collection, integration, and scaling, were carried out using the Bruker APEXII software. Absorption corrections were applied using SADABS. Structures were solved by direct methods using SHELXS and refined against *F*^2^ on all data by full-matrix least-squares with SHELXL-2014 or SHELXL-2018 using established refinement techniques. In this article, the visible figures of crystal structures are manipulated by software *Mercury 2023.3.0* and *CrystalExplorer 21.5*.

### 2.5 Bond Valence Sum Analysis

The oxidation states of the Cu ions in the metallopeptoids were determined by bond valence sum analysis^36,37^ with eq 1, according to the metal–ligand bond lengths measured from the single-crystal X-ray diffraction structure
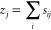
1where the *z*_*j*_ is the oxidation state of the cation *j* and *s*_*ij*_ is the bond valence between anion *i* and cation *j*. The value *s*_*ij*_ is determined by eq 2 shown below
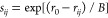
2where *r*_0_ and *B* are the constants depending on the nature of the bond between the cation *j* and anion *i*, and *r*_*ij*_ is the observed bond distance between cation *j* and anion *i* from single-crystal X-ray diffraction data.

### 2.6 Hirshfeld Surface Analysis

Hirshfeld surfaces analysis was calculated using the CIF files of the single crystals as the input file in the CrystalExplorer 21.5 package.^38,39^ For each point on the Hirshfeld surface, two distances are defined: *d*_*e*_ is the distance from the point to the nearest atom outside and *d*_*i*_ the distance to the nearest atom inside. The normalized contact distance, *d*_norm_, is given by the calculation based on both *d*_*e*_ and *d*_*i*_, and the van der Waals (vdW) radii of the atom, *r*^vdW^, which enables identification of the regions of particular importance to intermolecular interactions.^40^ Mathematically, *d*_norm_ is defined by eq 3 as follows (see details in refs (38–40))
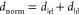
3where *d*_|*e*|_ and *d*_|*i*|_ are calculated by eqs 4 and 5
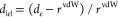
4
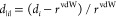
5In visible results of Hirshfeld surfaces, the red, white, and blue colors on the surface represent the *d*_norm_ with shorter, equal, and longer distance than vdW radii, respectively. The combination of *d*_*e*_ and *d*_*i*_ in the form of a 2D fingerprint plot provides a summary of intermolecular contacts in the corresponding crystal structure.^41^

## 3 Results and Discussion

### 3.1 Preparation of Metallopeptoids **1** and **2**

**Figure 2:**
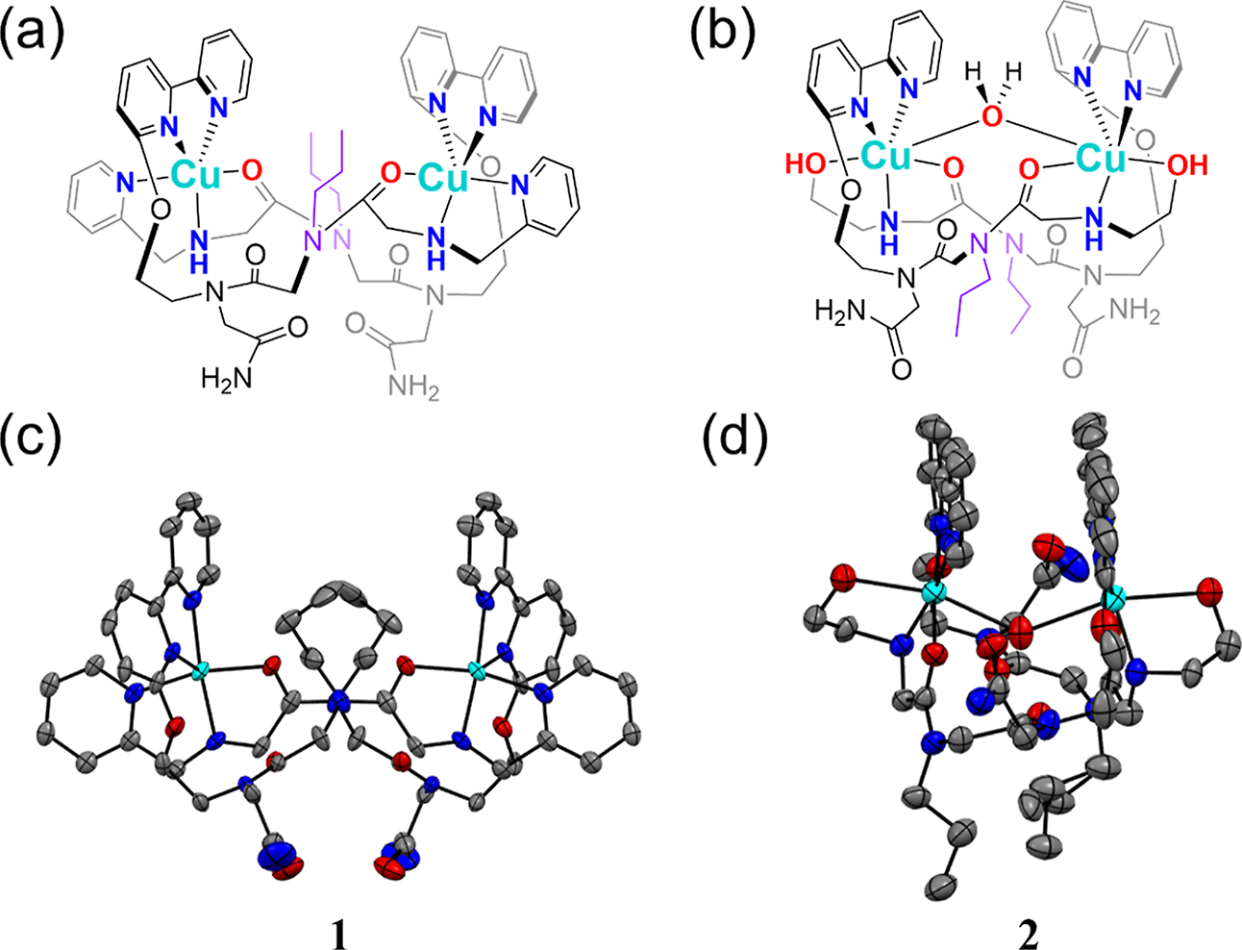
Molecular and X-ray structures of the metallopeptoids **1** (a,c) and **2** (b,d). The molecular views emphasize the propyl side chains as purple color. In the X-ray ORTEP (Oak Ridge Thermal–Ellipsoid Plot) views, thermal ellipsoids are drawn at the 50% probability level; gray: C, blue: N, red: O, and cyan: Cu; hydrogen atoms and guest molecules are omitted for clarity.

The designed peptoid ligands **L1** and **L2** (Figure 1) were prepared by the “sub-monomer” solid-phase synthesis, cleaved from solid support, and further purified by preparative HPLC.^42^ The obtained **L1** and **L2** (>95% purity) were characterized by ESI-MS, and the obtained masses were consistent with their calculated molecular weight (Figures S1 and S2). Peptoids **L1** and **L2** were dissolved in *n*-propanol and treated with Cu(ClO_4_)_2_·6H_2_O in ambient conditions.^19^ After 2 h of stirring, the precipitates were isolated by centrifugation, washed with *n*-propanol, and dried in air. Subsequently, the dry powders were redissolved in acetonitrile/water (the complex was dissolved in 0.5 mL of acetonitrile, and a few drops of water were added until a clear solution was obtained) and slowly crystallized with solvent evaporation. The crystals were characterized by ESI-MS showing a dominant mass of 1463 *m*/*z* (Figure S3), which was consistent with the single-crystal X-ray diffraction analysis, indicating the X-ray structure of the self-assembled duplex [Cu_2_(**L1**)_2_(ClO_4_)_3_]^+^ (**1**, Figures 2 and S4). Likewise, the characterization of the crystal derived from **L2** exhibited the formation of complex **2** (Figures 2 and S5), which has been reported previously.^13^ The comparison of the X-ray crystal data and structure refinement for **1** and **2** are presented in Table 1.

**Table 1:** Crystal Data and Structure Refinement for **1** and **2**

identification code	**1**	**2**
empirical formula	C_54_H_66_Cl_4_Cu_2_N_14_O_24_	C_48_H_69_Cl_4_Cu_2_N_13_O_27_
formula weight	1564.08	1529.07
temperature/K	100.15	200.15
crystal system	monoclinic	triclinic
space group	*C*2/*c*	*P*1̅
*a*/Å	29.6598(8)	13.800(2)
*b*/Å	19.3791(5)	15.635(2)
*c*/Å	11.2684(4)	16.505(2)
α/°	90	75.627(2)
β/°	95.388(3)	73.025(3)
γ/°	90	76.260(4)
volume/Å^3^	6448.2(3)	3246.6(8)
*Z*	4	2
ρ_calc_g/cm^3^	1.611	1.564
μ/mm^–1^	0.917	0.912
F(000)	3224.0	1580.0
crystal size/mm^3^	0.18 × 0.18 × 0.12	0.24 × 0.15 × 0.09
radiation	Mo Kα (λ = 0.71073)	Mo Kα (λ = 0.71073)
2Θ range for data collection/°	4.522–59.408	2.63–48.476
index ranges	–38 ≤ *h* ≤ 38, –26 ≤ *k* ≤ 25, –12 ≤ *l* ≤ 15	–15 ≤ *h* ≤ 15, –18 ≤ *k* ≤ 18, –19 ≤ *l* ≤ 19
reflections collected	26,811	29,538
independent reflections	7403 [*R*_int_ = 0.0505, *R*_sigma_ = 0.0480]	10,079 [*R*_int_ = 0.0856, *R*_sigma_ = 0.1252]
data/restraints/parameters	7403/462/464	10,079/990/928
goodness-of-fit on *F*^2^	1.044	1.000
final *R* indexes [*I* ≥ 2σ (*I*)]	*R*_1_ = 0.0514, *wR*_2_ = 0.1405	*R*_1_ = 0.0661, *wR*_2_ = 0.1626
final *R* indexes [all data]	*R*_1_ = 0.0794, *wR*_2_ = 0.1565	*R*_1_ = 0.1633, *wR*_2_ = 0.2183
largest diff. peak/hole/e Å^–3^	0.81/–0.55	1.05/–0.76

### 3.2 Molecular Structural Studies

The X-ray structure of **1** reveals a typical Cu-peptoid intermolecular complexation with two **L1** peptoids and two Cu ions forming a Cu_2_L_2_ duplex in which both Cu ions are coordinated in a square pyramidal geometry (Figure 2).^20^

**Figure 3:**
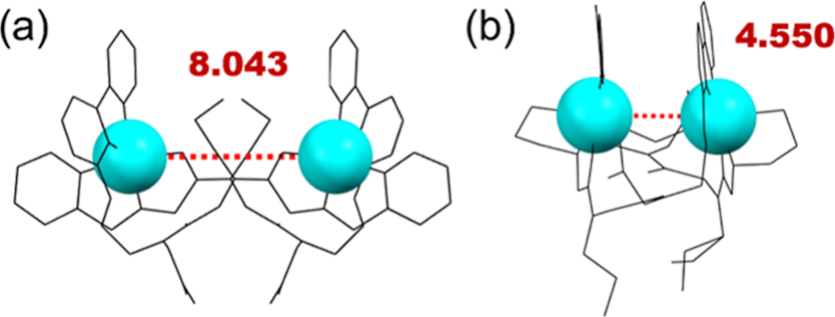
Wireframe style of **1** (a) and **2** (b), where the Cu ions are emphasized with space fill style and cyan color. The red dashed line and the values represent the intramolecular Cu···Cu distance.

In depth, each Cu ion coordinates to two N atoms from 2,2′-bipyridyl side chain in a peptoid **L1**; and one N atom from pyridine, one N atom from secondary amine, and one O atom from amide backbone in the other peptoid **L1**. These two Cu coordination environments (bond length and bond angle) exhibit complete identity and symmetry along a C2 axis (Tables S2 and S3, Figure S6). According to the bond length data of Cu-L, both Cu ions were assigned as Cu^2+^ in **1** by bond valence sum analysis (Table S1).^36,37^ Therefore, **1** contains a +4 charge as [Cu_2_(**L1**)_2_]^4+^. In parallel, **2** comprises the same folding of two peptoid ligands **L2** along a C2 symmetry axis, and both Cu ions were calculated to be Cu^2+^ by the same method. However, the Cu coordination environments in **2** are slightly different from each other due to different bond distance from the same location (Tables S4 and S5). Notably, a guest H_2_O molecule coordinates to both Cu atoms as a bridge, forming a distorted octahedral geometry in **2**, a phenomenon not observed in **1**. Typically, dinuclear Cu-peptoid features Cu···Cu distances ranging from 4.2 to 6.9 Å,^8,13,19,20^ and in most cases, a guest H_2_O molecule between two Cu ions results in a shorter Cu···Cu distance, as observed in **2** with 4.550 Å. However, the Cu···Cu distance of **1** exceeds the typical range, extending up to 8.043 Å (Figure 3).

Another interesting inspection was that the propyl side chains in **2** expand beyond the folding structure, whereas in **1**, these side chains intrude between the two Cu ions, therefore exerting steric hindrance and pushing the Cu ions apart.^43,44^ We expected that this difference is caused by the replacement of the ethanolic side chain from **2** to the pyridyl side chain in **1** at the N-terminal of the peptoid. Since the ethanolic side chain is hydrophilic and the pyridyl side chain is hydrophobic, we further assume that this side chain replacement affects the intra/intermolecular interactions when compared **1** to **2**, therefore the hydrophobic propyl side chains orientate either inside or outside the molecule,^41^ leading to drastic difference of Cu···Cu distance.

### 3.3 Inter/intramolecular Interactions of **1**

**Figure 4:**
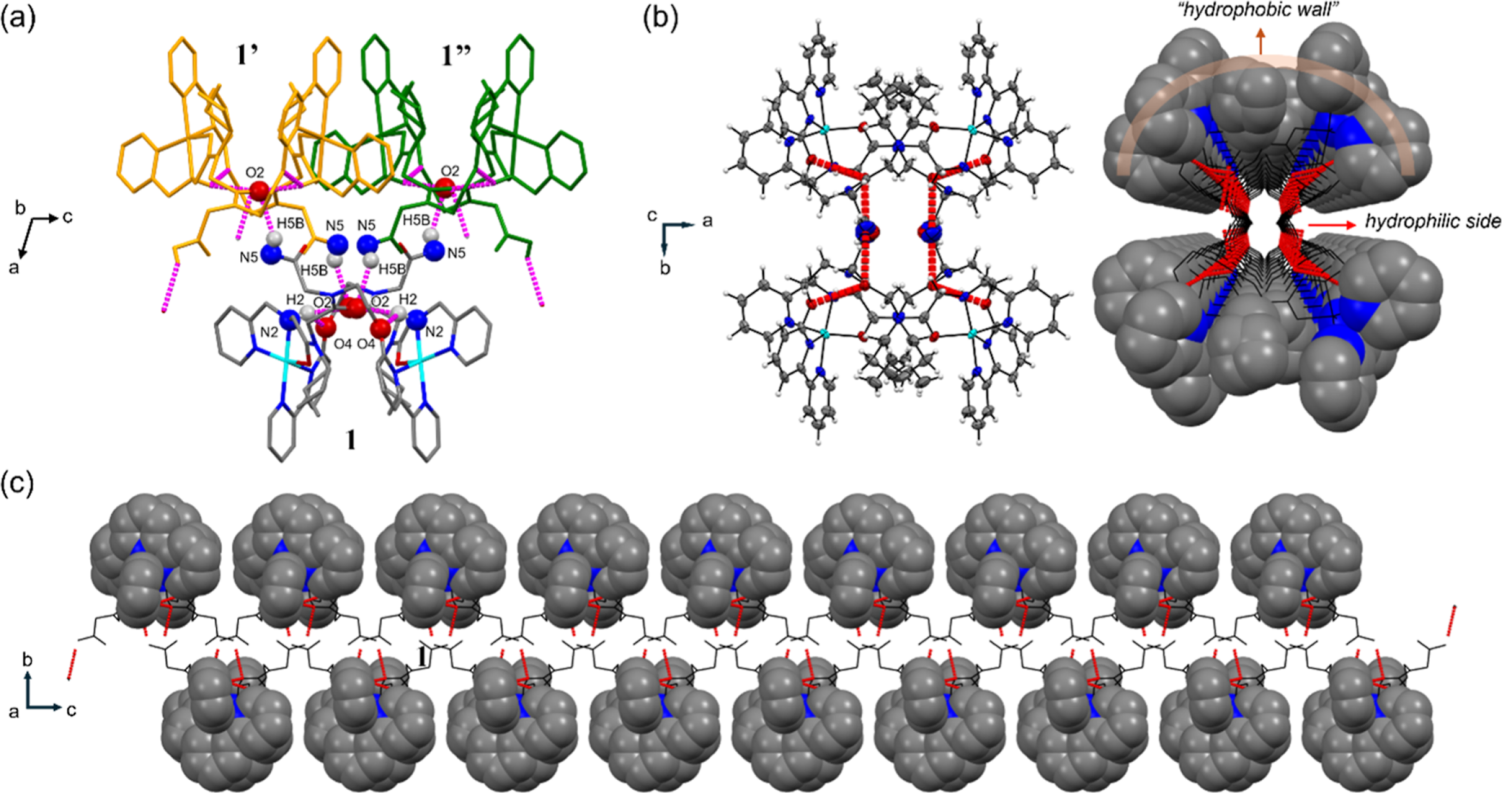
(a) Capped-sticks view of **1** and its intermolecular neighbors **1′** (yellow) and **1″** (green) along the *b*-axis; atoms that are related to H-bonds are labeled; H-bonds are marked with pink dashed lines. (b) ORTEP style (left) and space fill + wireframe style (right) of **1** along the *c*-axis; H-bonds are marked as red dashed lines; the hydrophobic elements are shown with space fill style and the hydrophilic elements are shown with wireframe style; the perspective view in this orientation displays a channel; (c) sequential intermolecular H-bonds (red dashed line) along the *a*-axis.

To gain deeper insights into the significant factors that affect the Cu···Cu distance, the intermolecular and intramolecular interactions were investigated by single-crystal X-ray structural and Hirshfeld surface analysis. Considering hydrogen bonds (H-bonds) inside the self-assembled structure, **1** shows two pairs of identical intramolecular H-bonds (in a total of 4 intramolecular H-bonds) for each Cu ion involving the donation of a proton H^+^ from the secondary amine (H2–N2) and its acceptance by O4 (N2–H2···O4, 2.473 Å) and O2 (N2–H2···O2, 2.053 Å) in the side chain backbone (Table 2). For the intermolecular interaction of **1** to others, four intermolecular H-bonds were found with two neighbors, **1′** and **1″** (Figure 4): (1) the amide at the C-terminal of **L1** in **1** donates a H^+^ to the O atom of amide in the proximal **1′** (N5–H5B···O2, 2.376 Å); (2) the O atom of amide accepts back a H^+^ from the amide at the C-terminal of the same neighbor **1′** (O2···H5B–N5, 2.376 Å); (3) similar to (1), a H^+^ of the amide at the C-terminal of the other **L1** in **1** is accepted by its nearby **1″** (N5–H5B···O2, 2.376 Å); (4) similar to (2), the O atom of amide from the other **L1** accepts a H^+^ from its nearby **1″** (O2···H5B–N5, 2.376 Å). The H-bond lengths and atom labels between **1** and **1′** are identical to that between **1** and **1″**. Interestingly, **1** has no H-bonds with guest molecules (e.g., H_2_O or ClO_4_^–^) and these four intermolecular H-bonds are all orientated in the same direction parallel to the *b*-axis. These orientated hydrogen bonds assist the molecules of **1** to proximate their neighbors in an organized order. Additionally, the π–π intermolecular interactions (ca. 3.270 Å) between the pyridyl side chains were also found to contribute to the organized order orientation (Figure S7). Ultimately, both H-bonds and aromatic stacking assist in generating a channel (Figure 4b,c).^45−47^

**Table 2:** Hydrogen (H–) Bond Data Summary of **1** and **2**

complex	**1**		**2**	
intramolecular	N2–H2···O4	2.473 Å	N7–H7···O3	2.297 Å
	N2–H2···O2	2.053 Å	N7–H7···O5	2.219 Å
	N2–H2···O4′	2.473 Å	O11–H11A···O3	1.968 Å
	N2–H2···O2′	2.053 Å	O11–H11B···O9	2.127 Å
			N1–H1A···O8	2.223 Å
			N1–H1A···O9	2.142 Å
intermolecular	N5–H5B···O2	2.376 Å	N5–H5A···N13	2.249 Å
	N5–H5B···O2′	2.376 Å	N5–H5B···O25	2.912 Å
	N5–H5B···O2″	2.376 Å	N5–H5B···O6	2.235 Å
	N5–H5B···O2‴	2.376 Å	O6–H6···O10	1.842 Å
			O1–H1···013	1.953 Å
			N10–H10A···N13	2.310 Å
			N10–H10A···O23	2.659 Å
			N10–H10B···O20	2.282 Å
			N10–H10B···O15	2.957 Å
			O6–H6···O10′	1.842 Å
			N5–H5B···O6′	2.235 Å

**Figure 5:**
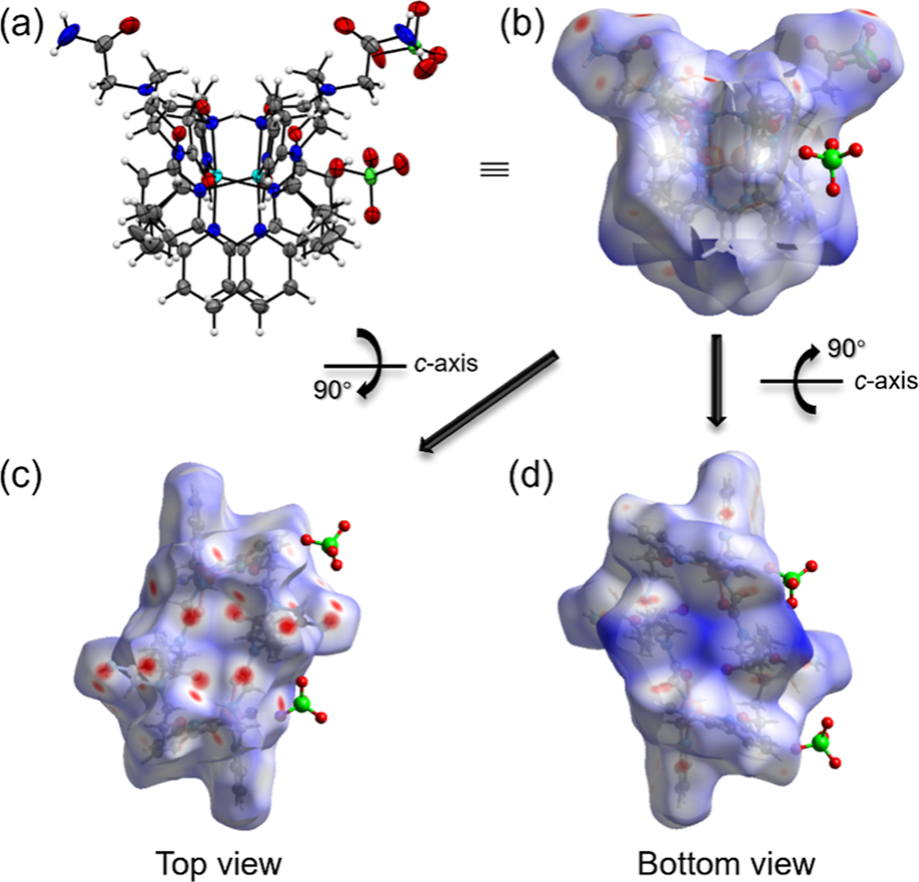
(a) ORTEP view and (b) Hirshfeld surface of **1** at the same orientation; (c) top view and (d) bottom view of **1** by rotating (b) view to 90° in opposite direction.

Notably, all the side chains of **L1** (2,2′-bipyridyl, propyl, and pyridyl groups) create a “hydrophobic wall” outside the channel owing to their intrinsic hydrophobicity and π–π interactions, while the inside is hydrophilic including all the H-bonds formed by the peptoid backbone.^31,48,49^ This hydrophobic/hydrophilic division is further supported by Hirshfeld surface analysis of the surface of crystal structure **1** calculated in *CrystalExplorer* package,^38,39^ and the results are shown in Figure 5. Figure 5a,b displays the ORTEP view and the surface view of **1**, respectively, at the same orientation, and Figure 5c,d shows the top view and bottom view of the surface of **1** after the according rotation from Figure 5b. Based on our structural analysis, the top view (Figure 5c) is consistent with the observation that the hydrophilic side contains all the intermolecular H-bonds within the terminal amide and amine on the peptoid backbone; thereby, the bottom view (Figure 5d) demonstrates the “hydrophobic wall” where the 2,2′-bipyridyl, propyl, and pyridyl side chains are located. Multiple red spots that represent intermolecular interaction with short vdW radii are observed on the Hirshfeld surface of the hydrophilic side (top view) due to the assigned intermolecular H-bonds.^50^ In the fingerprint plot of this Hirshfeld surface (Figure S8), H-bonds dominate at 39.7% (where 35.8% for inside and outside H; 3.9% for inside and outside H) versus various intermolecular interactions. In contrast, the “hydrophobic wall” in the bottom view displays a blue color, where the spots are far away from the neighboring molecules, indicating intermolecular interaction with long vdW radii in these areas.

### 3.4 Comparison between **1** and **2**

**Figure 6:**
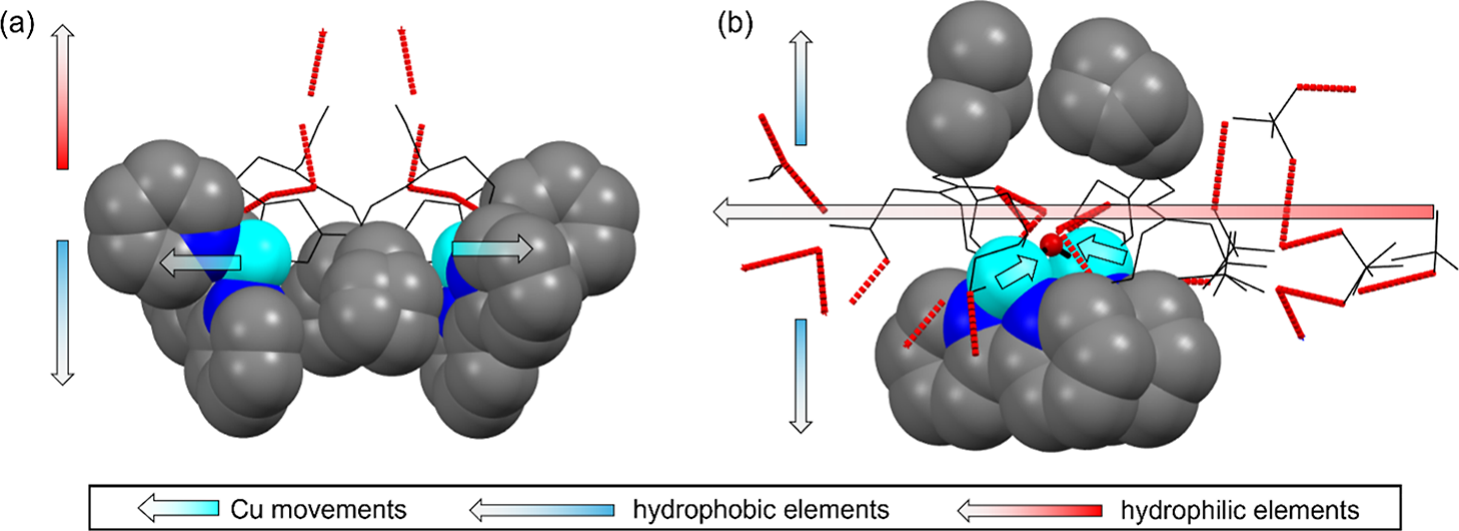
Space fill and wireframe mix styles of (a) **1** and (b) **2** for emphasizing the hydrophilic H-bonds (red dashed line) and hydrophobic elements (propyl, pyridyl, and 2,2′-bipyridyl side chains).

We have demonstrated that the self-assembled structure of **1** is characterized by a division into a “hydrophobic wall” and a hydrophilic interior, which form a channel via H-bonds. In the structure of **2**, the H_2_O bridge between the Cu ions and the alternative ethanolic side chains results in increased number of intramolecular and intermolecular H-bonds, compared to **1** (Table 2). In addition, the ethanolic side chains (O1–H1 and O6–H6) and the amide groups at the C-terminal (N10–H10 and N5–H5) build extra H-bonds with guest molecules such as ClO_4_^–^ and CH_3_CN. Consequently, each complex **2** directly binds to only one more complex **2** by H-bonds forming an intermolecular dimer (Figure S9), which cannot assemble further with more molecules or dimers of **2** via H-bonds as these are occupied by host–guest interactions.^51^ This suggests that the introduction of the ethanolic side chain increases the number of H-bonds by up to 48.1%, as indicated from the fingerprint of the Hirshfeld surface (Figure S10), but disrupts the channel formation observed in **1**. Furthermore, the orientation of the van der Waals forces^52^ differs between **1** and **2**. The H-bonds in **1** face upward (red arrow in Figure 6), while the hydrophobic parts including the propyl side chains face downward (blue arrow) on the opposite side. This, along with all the side chains of **L1** further generate the “hydrophobic wall”. The propyl side chains create steric hindrance between the Cu ions, enlarging the Cu···Cu distance up to 8.043 Å. In contrast, in **2**, the ethanolic side chains contribute to the horizontal orientation of the H-bonds (in the view of Figure 6), allowing the propyl side chains to swing upward against the 2,2′-bipyridyl. In addition, a much shorter Cu···Cu distance of 4.550 Å in **2** allows a guest H_2_O molecule to interact with both Cu ions as a bridging molecule between them. This phenomenon has also been demonstrated in other Cu-peptoid crystals reported by our group.^19,20^

## 4 Conclusions

In summary, we prepared and characterized a new dinuclear Cu-peptoid, **1**, from the peptoid ligand **L1** consisting of hydrophobic 2,2′-bipyridyl, propyl, and pyridyl side chains. The X-ray crystal structure and ESI-MS indicated that **1** is a self-assembled dinuclear structure exhibiting a long Cu···Cu distance of 8.043 Å that exceeds the Cu···Cu distance of similar dinuclear Cu-peptoids, which is typically in the range of 4.2–6.9 Å. Based on molecular structural analysis and comparison with a control complex **2**, in which the pyridyl group was replaced by an ethanolic side chain, we realized that the pyridyl side chain in **L1** impacts the orientation of the H-bonds, therefore preserving the hydrophobic propyl side chain in between two Cu ions, generating steric hindrance to elongate the Cu···Cu distance. This study also unveils the importance of side chain selection and intramolecular metal–metal distance tuning and controlling for metallopeptoid designs. With that, we envision that the range of Cu···Cu distance of dinuclear Cu peptoids is much broader than the current known data, and this can expand the potential of applications for metallopeptoids having flexible and tunable macrostructure. For example, supramolecular interactions of different Cu···Cu distances can response to different wavelengths of luminescence, therefore addressing the need of color for imaging application.^27,28,53,54^ In addition, Cu···Cu distance can be a key factor for catalysis, such as CO_2_, O_2_, and N_2_ reductions, toward the production of selective products.^21,23,55^

## Supporting Information Available

Analytical HPLC and ESI-MS spectra of **L1** and**1**; bond valence sum data, Hirshfeld surface, and fingerprint plot of **1** and **2**; hydrogen bond analysis of **2**; and crystal structure data of **1** and **2** (PDF)
