# Supplementary material for: Unique Crystal Structure of a Self-Assembled Dinuclear Cu Peptoid Reveals an Unusually Long Cu···Cu Distance
Source: ACS Omega. 2024 Sep 26;9(40):42002–9. doi: 10.1021/acsomega.4c06987 (PMC11465249; doi:10.1021/acsomega.4c06987)
Supplement: Supplementary file 1 — ao4c06987_si_001.pdf [file ao4c06987_si_001.pdf]

Supporting Information

Unique Crystal Structure of a Self-Assembled Dinuclear Cu Peptoid Reveals an Unusually Long Cu---Cu Distance

*Guilin Ruan, Natalia Fridman, Galia Maayan**

Schulich Faculty of Chemistry, Technion−Israel Institute of Technology, Haifa, 32000, Israel.

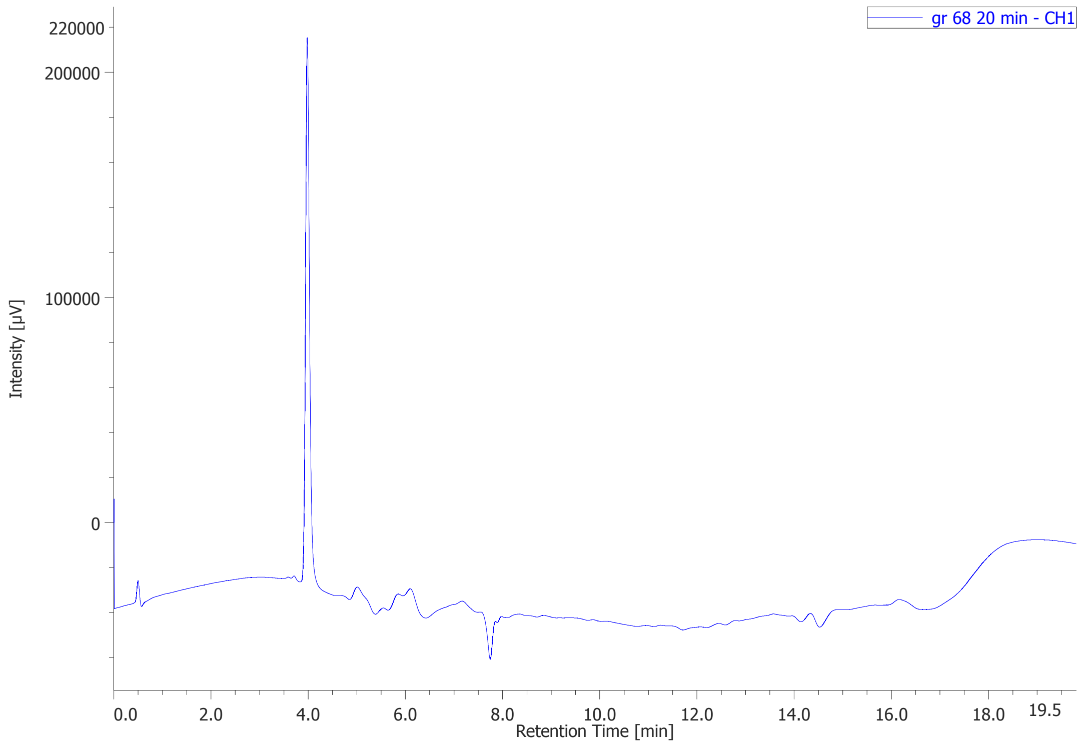

Figure S1. HPLC of the peptoid **L1**.

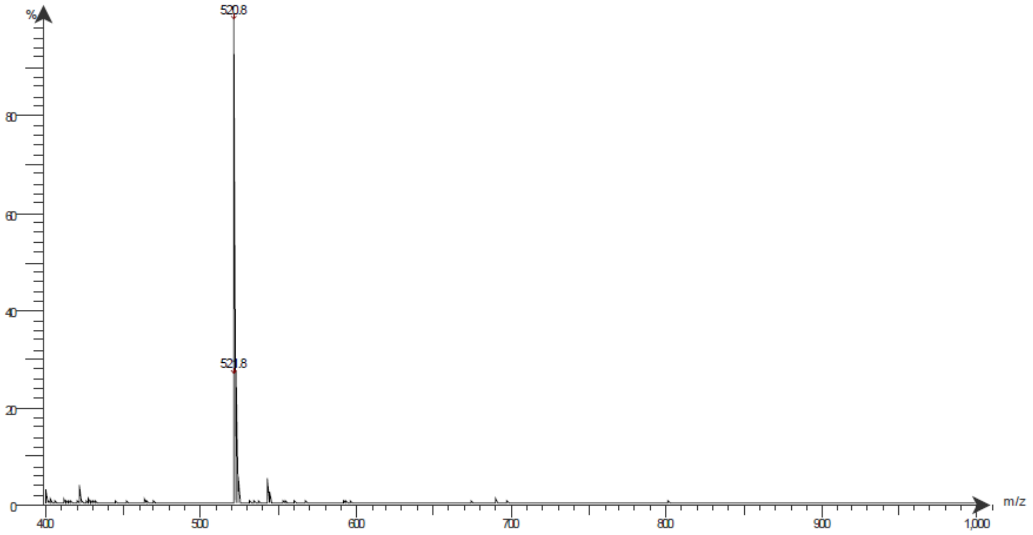

Figure S2. ESI-MS of the peptoid **L1**.

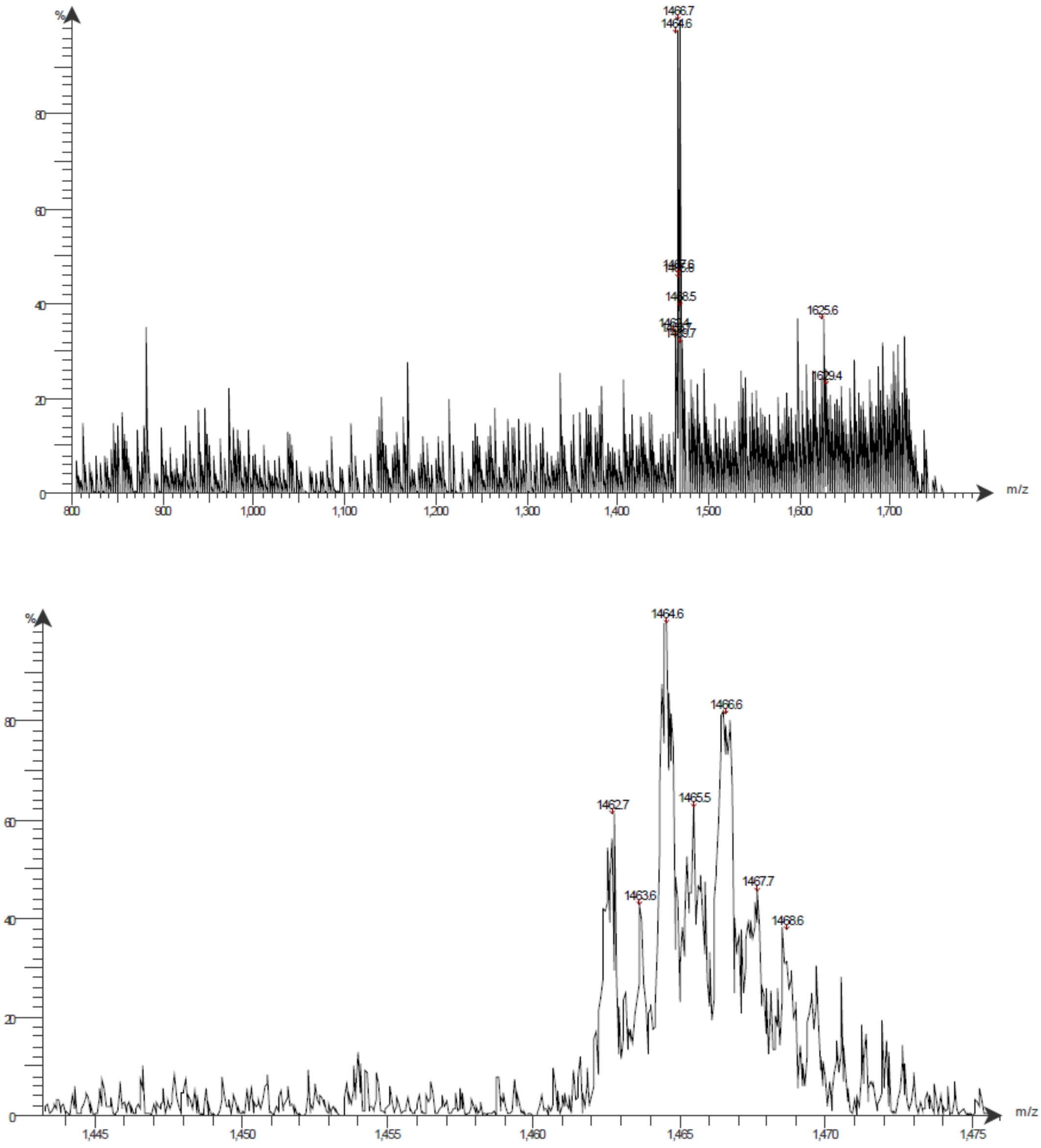

Figure S3. ESI-MS of the metallopeptoid **1**. Up: range from 800 to 1800 m/z; down: narrow view of $[Cu_2(\mathbf{L1})_2(ClO_4)_3]^+$ (1464 m/z) in the range of 1445 to 1475 m/z with clear isotope distribution of Cu and Cl.

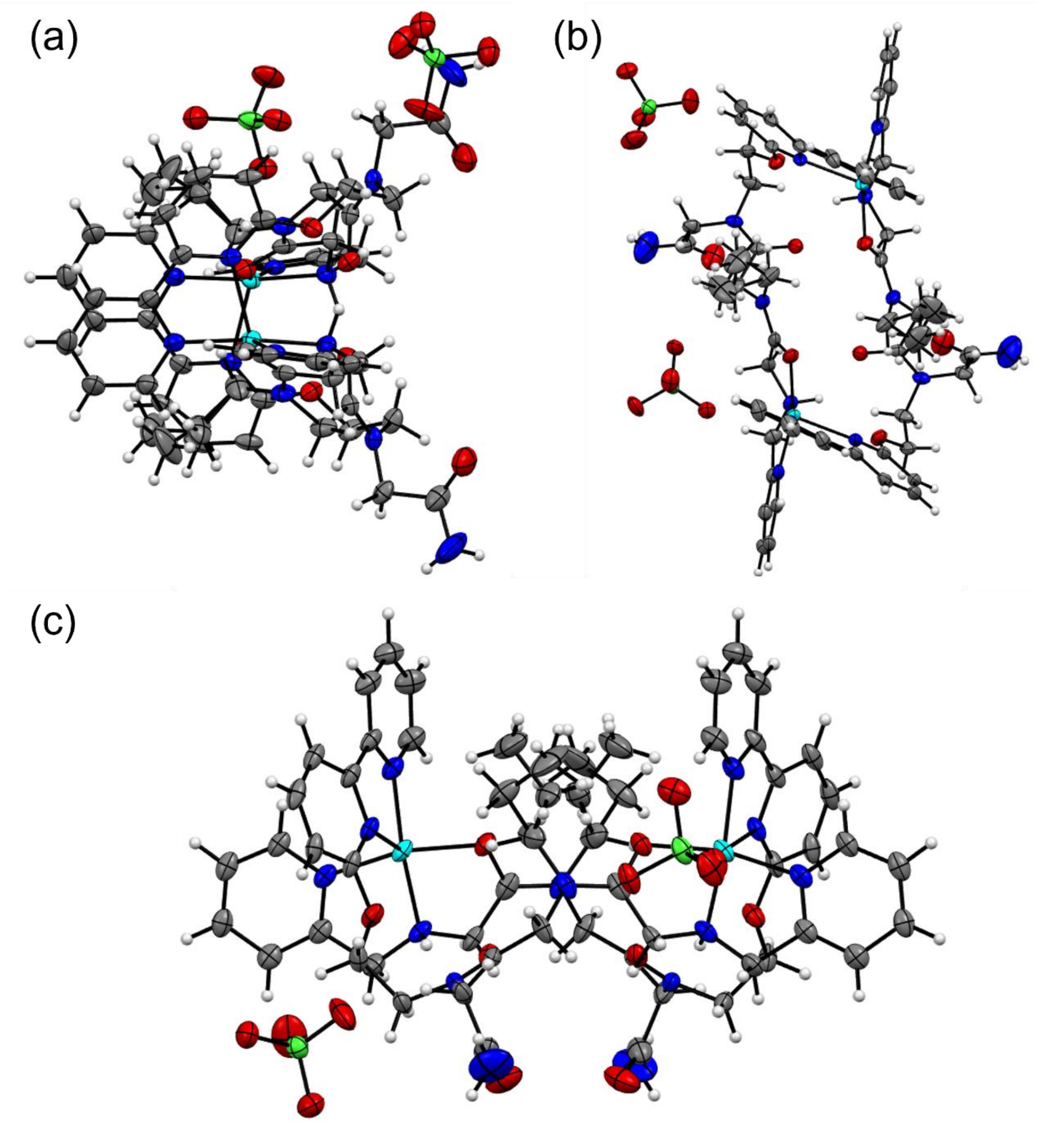

Figure S4. Crystal structure of the metallopeptoid **1**. (a) a-axis; (b) b-axis; (c) c-axis.

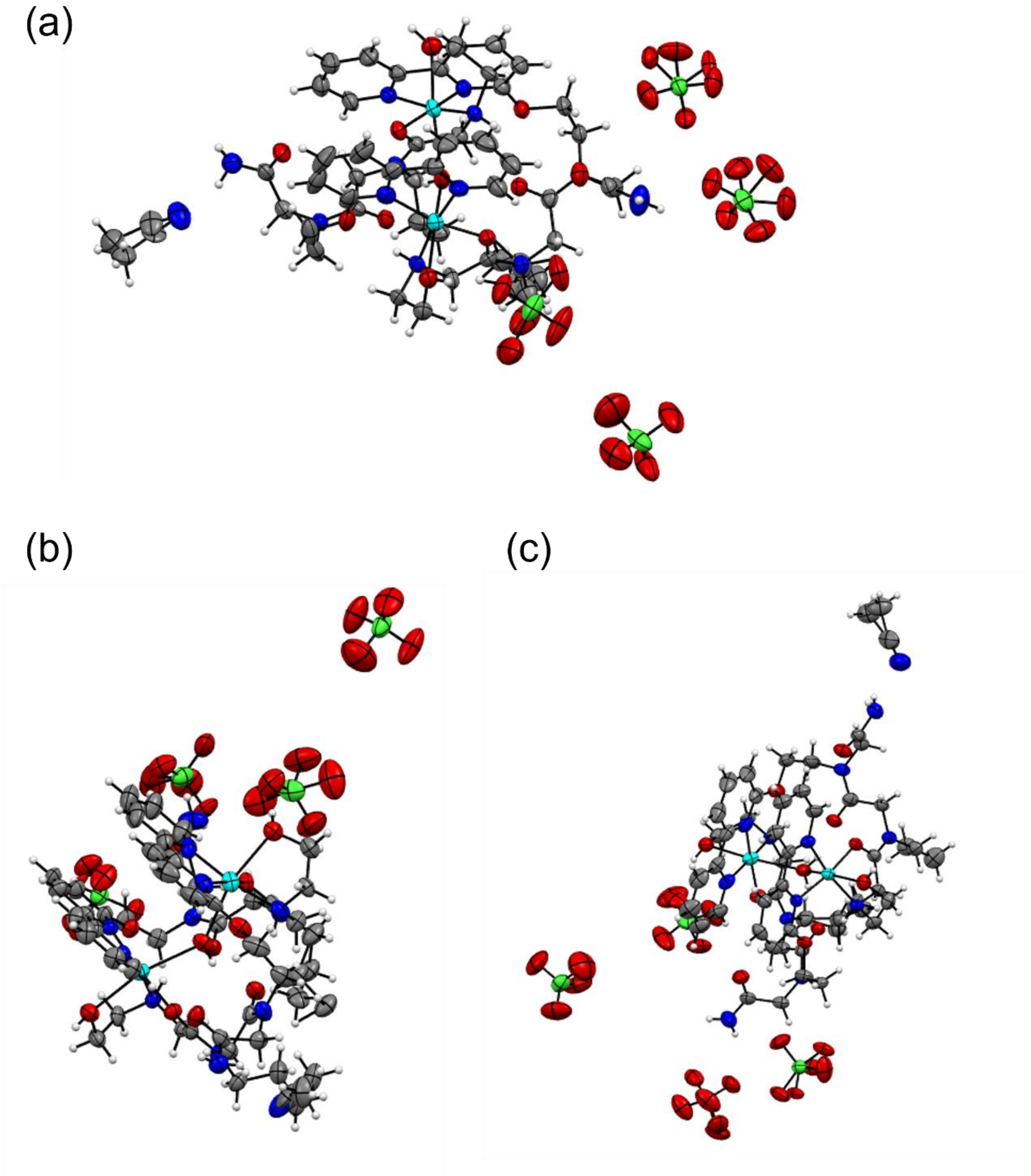

Figure S5. Crystal structure of the metallopeptoid **1**. (a) a-axis; (b) b-axis; (c) c-axis.

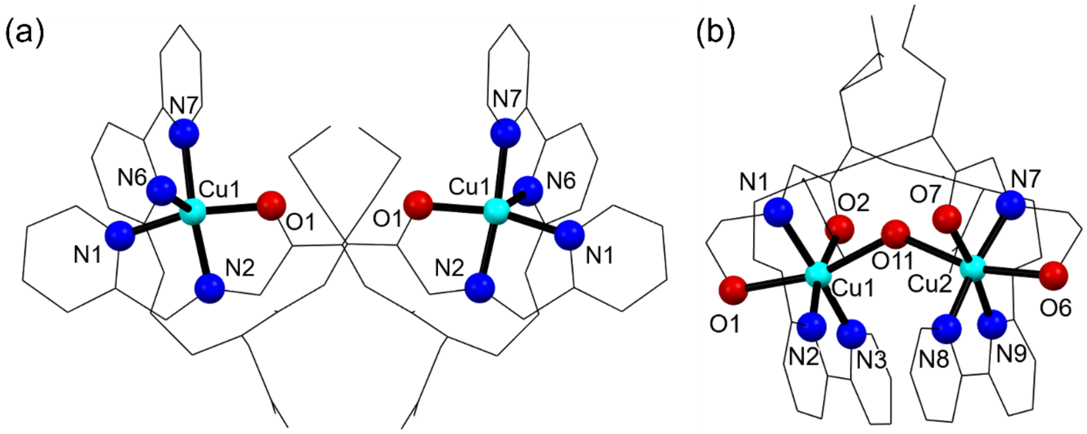

Figure S6. Coordination and geometries of Cu centers in **1** and **2**.

(a) In **1**: Cu1-N1: 2.007 Å; Cu1-N2: 1.990 Å; Cu1-N6: 2.262 Å; Cu1-N7: 1.993 Å; Cu1-O1: 1.996 Å.

(b) In **2**: Cu1-N1: 2.020 Å; Cu1-N2: 2.003 Å; Cu1-N3: 1.966 Å; Cu1-O1: 2.422 Å; Cu1-O2: 1.942 Å, Cu1-O11: 2.481 Å; Cu2-N7: 2.016 Å; Cu2-N8: 1.969 Å; Cu2-N9: 2.032 Å; Cu2-O6: 2.331 Å, Cu2-O7: 1.979 Å; Cu2-O11: 2.461 Å.

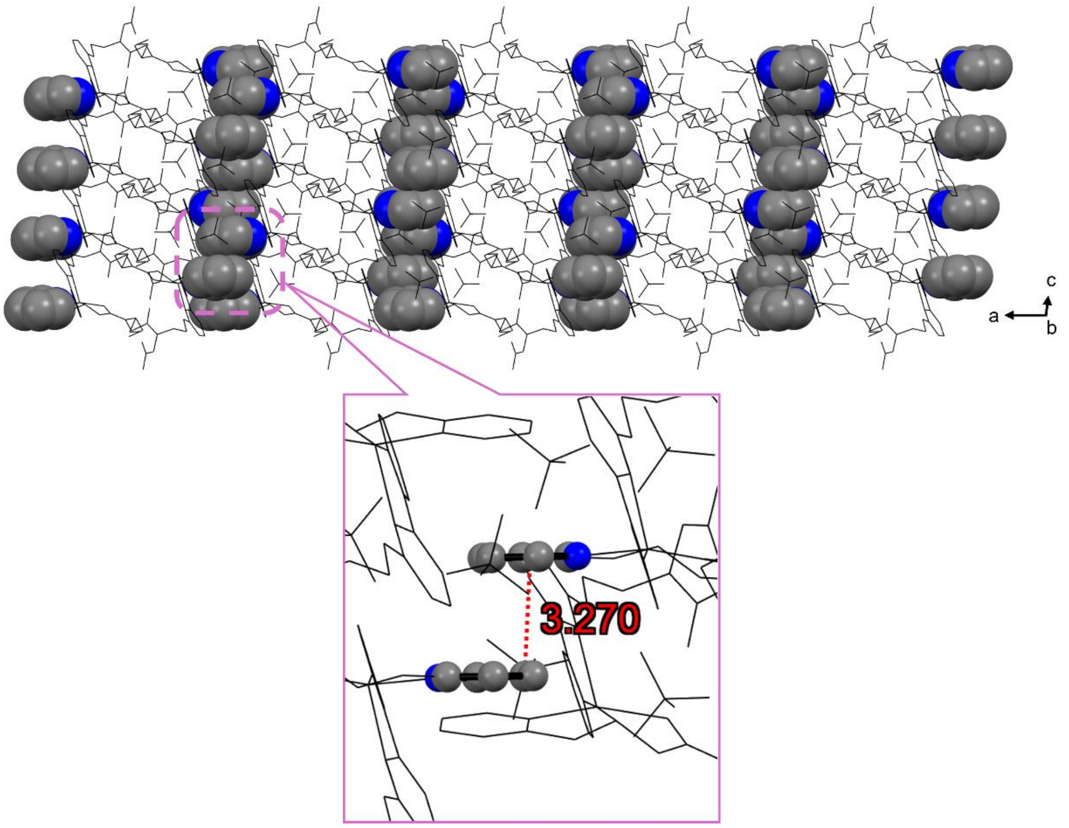

Figure S7. Aromatic stacking of **1** in the view along b-axis.

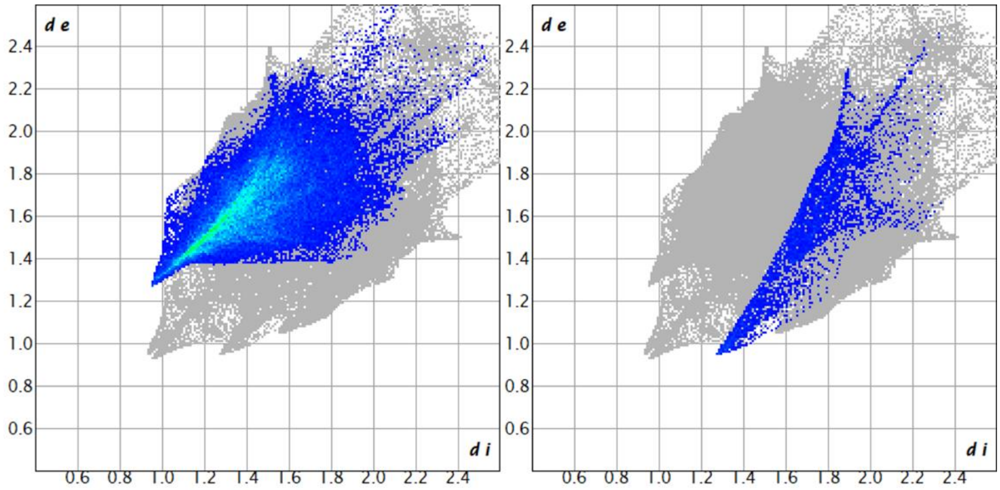

Figure S8. Fingerprint of the Hirshfeld surface of **1** corresponding to H-bonds that dominates 39.7 %, where 35.8 % for inside O and outside H (left) and 3.9 % for inside O and outside H (right).

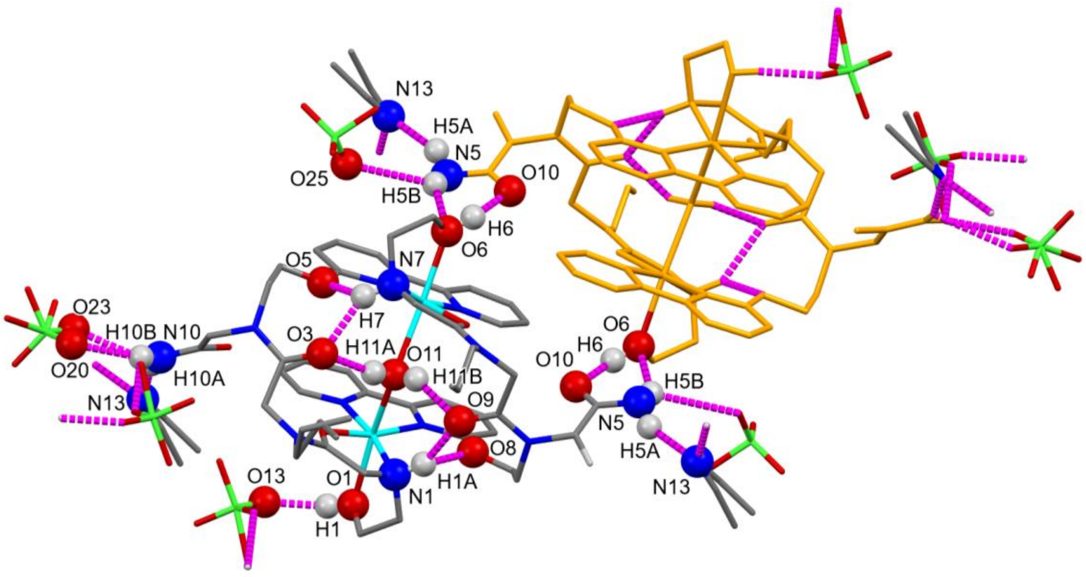

Figure S9. H-bonds of between complex **2** and its neighbor (yellow), forming an intermolecular dimer.

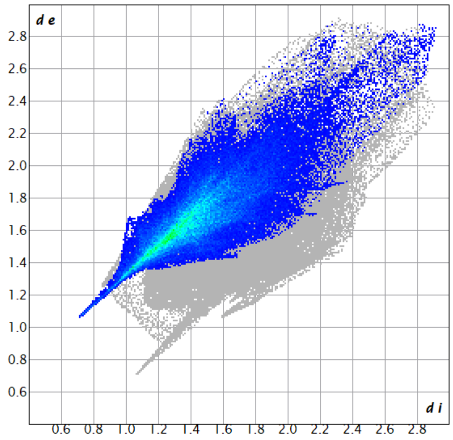

Figure S10. Fingerprint of the Hirshfeld surface of **2** corresponding to H-bonds that dominates 48.1 %, where 2.8 % for inside O and outside H (left) and 45.3 % for inside O and outside H (right).

Table S1. Bond valence sum data for **1** and **2**.

Crystal	M-L bond	r_{ij}	r_0	s_{ij}	BVS
	Cu1-O1	1.996(2)	1.649	0.391473	2.013
	Cu1-N1	2.006(3)	1.713	0.452987	
**1**	Cu1-N6	2.262(3)	1.713	0.226778	
	Cu1-N7	1.993(3)	1.713	0.469186	
	Cu1-N2	1.990(3)	1.713	0.473005	
	Cu1-O1	2.421(5)	1.649	0.124122	2.287
	Cu1-O2	1.942(4)	1.649	0.452987	
**2**	Cu1-N1	2.020(6)	1.713	0.436167	
	Cu1-N2	2.004(6)	1.713	0.455442	
	Cu1-N3	1.966(6)	1.713	0.504704	
	Cu1-O11	2.077[1]	1.649	0.31442	
	Cu2-O6	2.331(5)	1.649	0.158303	2.041
	Cu2-O7	1.979(5)	1.649	0.40988	
**2**	Cu2-N7	2.016(5)	1.713	0.440908	
	Cu2-N8	1.969(6)	1.713	0.500628	
	Cu2-N9	2.032(6)	1.713	0.422248	
	Cu2-O11	2.461[1]	1.649	0.111374	

Table S2 Bond Lengths for **1**.

**Atom**	**Atom**	**Length/Å**	**Atom**	**Atom**	**Length/Å**
Cu1	O1	1.996(2)	N2	C6	1.483(4)
Cu1	N1	2.006(3)	N3	C8	1.321(4)
Cu1	N6[1]	2.262(3)	N3	C9	1.456(4)
Cu1	N7[1]	1.993(3)	N3	C25	1.458(5)
Cu1	N2	1.990(3)	O3	C12	1.212(5)
Cl2	O10	1.438(3)	C20	C19	1.469(5)
Cl2	O9	1.436(3)	C20	C21	1.388(5)
Cl2	O11	1.432(3)	C10	C9	1.537(4)
Cl2	O12	1.417(3)	C19	C18	1.380(4)
Cl1	O6	1.443(2)	C16	C15	1.392(4)
Cl1	O8	1.447(3)	C16	C17	1.368(5)
Cl1	O5	1.437(3)	C1	C2	1.378(5)
Cl1	O7	1.431(3)	C5	C4	1.379(5)
O1	C8	1.263(4)	C5	C6	1.511(4)
O4	C15	1.347(4)	C7	C8	1.508(5)
O4	C14	1.449(4)	C18	C17	1.384(5)
O2	C10	1.238(4)	C2	C3	1.375(5)
N4	C10	1.343(4)	C4	C3	1.400(4)
N4	C13	1.470(4)	C13	C14	1.507(5)
N4	C11	1.439(4)	C11	C12	1.531(5)
N1	C1	1.348(4)	C21	C22	1.378(5)
N1	C5	1.342(4)	C24	C23	1.368(5)
N6	Cu1[1]	2.262(3)	C23	C22	1.371(5)

Table S2 Bond Lengths for **1**.

**Atom**	**Atom**	**Length/Å**	**Atom**	**Atom**	**Length/Å**
N6	C19	1.359(4)	C12	N5	1.348(6)
N6	C15	1.332(4)	C25	C26	1.572(8)
N7	Cu1[1]	1.993(3)	C25	C26A	1.526(12)
N7	C20	1.359(4)	C26	C27	1.503(9)
N7	C24	1.346(4)	C26A	C27A	1.45(2)
N2	C7	1.475(4)			

Table S3 Bond Angles for **1**.

Atom	Atom	Atom	Angle/	Atom	Atom	Atom	Angle/
O1	Cu1	N1	158.38(10)	N7	C20	C19	115.8(3)
O1	Cu1	N6[1]	111.63(9)	N7	C20	C21	121.0(3)
N1	Cu1	N6[1]	88.43(10)	C21	C20	C19	123.2(3)
N7[1]	Cu1	O1	91.98(10)	O2	C10	N4	123.5(3)
N7[1]	Cu1	N1	100.66(10)	O2	C10	C9	119.7(3)
N7[1]	Cu1	N6[1]	76.97(10)	N4	C10	C9	116.8(3)
N2	Cu1	O1	81.87(10)	N6	C19	C20	114.8(3)
N2	Cu1	N1	83.70(11)	N6	C19	C18	121.6(3)
N2	Cu1	N6[1]	110.11(10)	C18	C19	C20	123.5(3)
N2	Cu1	N7[1]	171.93(11)	C17	C16	C15	117.6(3)
O9	Cl2	O10	109.03(16)	N1	C1	C2	122.1(3)
O11	Cl2	O10	109.75(17)	N1	C5	C4	122.5(3)
O11	Cl2	O9	109.11(19)	N1	C5	C6	116.8(3)
O12	Cl2	O10	109.59(19)	C4	C5	C6	120.7(3)
O12	Cl2	O9	109.6(2)	N2	C7	C8	108.1(2)
O12	Cl2	O11	109.8(2)	O1	C8	N3	121.0(3)
O6	Cl1	O8	108.76(15)	O1	C8	C7	119.2(3)
O5	Cl1	O6	109.55(15)	N3	C8	C7	119.8(3)
O5	Cl1	O8	109.80(17)	O4	C15	C16	125.2(3)
O7	Cl1	O6	110.24(17)	N6	C15	O4	111.7(3)
O7	Cl1	O8	108.42(17)	N6	C15	C16	123.0(3)

Table S3 Bond Angles for **1**.

Atom	Atom	Atom	Angle/	Atom	Atom	Atom	Angle/
O7	Cl1	O5	110.05(18)	C19	C18	C17	118.5(3)
C8	O1	Cu1	112.3(2)	C3	C2	C1	118.8(3)
C15	O4	C14	118.8(3)	C5	C4	C3	117.9(3)
C10	N4	C13	119.6(3)	N2	C6	C5	110.8(3)
C10	N4	C11	124.7(2)	N4	C13	C14	109.2(3)
C11	N4	C13	115.7(2)	N3	C9	C10	111.0(3)
C1	N1	Cu1	126.5(2)	O4	C14	C13	105.8(2)
C5	N1	Cu1	114.5(2)	N4	C11	C12	110.0(3)
C5	N1	C1	118.8(3)	C2	C3	C4	119.8(3)
C19	N6	Cu1[1]	110.1(2)	C22	C21	C20	119.3(3)
C15	N6	Cu1[1]	127.5(2)	C16	C17	C18	120.7(3)
C15	N6	C19	118.5(3)	N7	C24	C23	122.1(3)
C20	N7	Cu1[1]	119.5(2)	C24	C23	C22	119.6(4)
C24	N7	Cu1[1]	121.5(2)	C23	C22	C21	119.2(3)
C24	N7	C20	118.7(3)	O3	C12	C11	122.9(4)
C7	N2	Cu1	108.6(2)	O3	C12	N5	123.3(4)
C7	N2	C6	113.4(2)	N5	C12	C11	113.5(4)
C6	N2	Cu1	112.38(19)	N3	C25	C26	105.2(4)
C8	N3	C9	122.4(3)	N3	C25	C26A	134.5(6)
C8	N3	C25	120.7(3)	C27	C26	C25	106.6(6)
C9	N3	C25	116.3(3)	C27A	C26A	C25	107.1(11)

Table S4 Bond Lengths for **2**.

Atom	Atom	Length/Å	Atom	Atom	Length/Å
Cu_1	O_1	2.421(5)	C_1	C_2	1.502(11)
Cu_1	O_2	1.942(4)	C_3	C_4	1.515(9)
Cu_1	N_1	2.020(6)	C_5	C_6	1.461(14)
Cu_1	N_2	2.004(6)	C_5	C_{6A}	1.548(18)
Cu_1	N_3	1.966(6)	C_6	C_7	1.4998(11)
Cu_2	O_6	2.331(5)	C_{6A}	C_{7A}	1.5001(11)
Cu_2	O_7	1.979(5)	C_8	C_9	1.518(11)
Cu_2	N_7	2.016(5)	C_{10}	C_{11}	1.530(11)
Cu_2	N_8	1.969(6)	C_{12}	C_{13}	1.506(10)
Cu_2	N_9	2.032(6)	C_{14}	C_{15}	1.383(10)
Cl_3	O_{20}	1.434(6)	C_{15}	C_{16}	1.380(11)
Cl_3	O_{20A}	1.16(4)	C_{16}	C_{17}	1.378(10)
Cl_3	O_{21}	1.434(8)	C_{17}	C_{18}	1.361(10)
Cl_3	O_{21A}	1.58(3)	C_{18}	C_{19}	1.483(9)
Cl_3	O_{22}	1.418(7)	C_{19}	C_{20}	1.401(10)
Cl_3	O_{23}	1.480(8)	C_{20}	C_{21}	1.374(10)
O_1	C_1	1.430(9)	C_{21}	C_{22}	1.363(11)
O_2	C_4	1.249(8)	C_{22}	C_{23}	1.385(10)
O_3	C_9	1.235(8)	C_{24}	C_{25}	1.486(10)
O_4	C_{11}	1.224(9)	C_{26}	C_{27}	1.512(9)

Table S4 Bond Lengths for **2**.

Atom	Atom	Length/Å	Atom	Atom	Length/Å
O5	C13	1.457(7)	C28	C29	1.498(11)
O5	C14	1.352(9)	C29	C30	1.537(12)
O6	C25	1.440(9)	C31	C32	1.521(11)
O7	C27	1.261(8)	C33	C34	1.530(11)
O8	C36	1.431(9)	C35	C36	1.497(13)
O8	C37	1.356(10)	C37	C38	1.399(11)
O9	C32	1.219(8)	C38	C39	1.333(14)
O10	C34	1.228(9)	C39	C40	1.399(13)
N1	C2	1.483(8)	C40	C41	1.376(11)
N1	C3	1.520(9)	C41	C42	1.472(11)
N2	C37	1.344(10)	C42	C43	1.371(11)
N2	C41	1.371(10)	C43	C44	1.387(12)
N3	C42	1.354(9)	C44	C45	1.366(12)
N3	C46	1.348(9)	C45	C46	1.384(12)
N4	C32	1.365(8)	Cl1	O12	1.520(11)
N4	C33	1.465(9)	Cl1	O12A	1.67(2)
N4	C35	1.458(10)	Cl1	O13	1.421(6)
N5	C34	1.335(9)	Cl1	O14	1.356(7)
N6	C27	1.312(9)	Cl1	O15	1.382(8)
N6	C28	1.504(9)	Cl2	O16	1.465(12)
N6	C31	1.457(8)	Cl2	O16A	1.30(3)
N7	C24	1.501(8)	Cl2	O17	1.436(6)
N7	C26	1.484(8)	Cl2	O18	1.404(5)

Table S4 Bond Lengths for **2**.

**Atom**	**Atom**	**Length/Å**	**Atom**	**Atom**	**Length/Å**
N_8	C_{19}	1.348(9)	Cl_2	O_{19}	1.411(9)
N_8	C_{23}	1.351(8)	Cl_2	O_{19A}	1.58(3)
N_9	C_{14}	1.327(8)	Cl_4	O_{24}	1.353(7)
N_9	C_{18}	1.376(9)	Cl_4	O_{25}	1.353(7)
N_{10}	C_{11}	1.308(10)	Cl_4	O_{26}	1.530(10)
N_{11}	C_9	1.358(8)	Cl_4	O_{27}	1.376(6)
N_{11}	C_{10}	1.459(9)	N_{13}	C_{47}	1.110(18)
N_{11}	C_{12}	1.456(9)	N_{13}	C_{47A}	1.107(14)
N_{12}	C_4	1.325(8)	C_{47}	C_{48}	1.48(2)
N_{12}	C_5	1.454(10)	C_{47A}	C_{48A}	1.48(2)
N_{12}	C_8	1.462(8)			

Table S5 Bond Angles for **2**.

Atom	Atom	Atom	Angle/	Atom	Atom	Atom	Angle/
O_2	Cu_1	O_1	93.39(19)	O_4	C_{11}	C_{10}	121.5(7)
O_2	Cu_1	N_1	83.7(2)	N_{10}	C_{11}	C_{10}	114.2(7)
O_2	Cu_1	N_2	168.3(2)	N_{11}	C_{12}	C_{13}	114.3(7)
O_2	Cu_1	N_3	90.3(2)	O_5	C_{13}	C_{12}	107.8(6)
N_1	Cu_1	O_1	77.9(2)	O_5	C_{14}	C_{15}	124.3(6)
N_2	Cu_1	O_1	96.1(2)	N_9	C_{14}	O_5	112.1(6)
N_2	Cu_1	N_1	105.0(3)	N_9	C_{14}	C_{15}	123.6(7)
N_3	Cu_1	O_1	94.8(2)	C_{16}	C_{15}	C_{14}	117.7(7)
N_3	Cu_1	N_1	170.2(3)	C_{17}	C_{16}	C_{15}	119.8(8)
N_3	Cu_1	N_2	82.2(3)	C_{18}	C_{17}	C_{16}	119.5(8)
O_7	Cu_2	O_6	92.5(2)	N_9	C_{18}	C_{19}	113.8(7)
O_7	Cu_2	N_7	83.2(2)	C_{17}	C_{18}	N_9	121.6(6)
O_7	Cu_2	N_9	172.8(2)	C_{17}	C_{18}	C_{19}	124.6(7)
N_7	Cu_2	O_6	78.4(2)	N_8	C_{19}	C_{18}	115.1(6)
N_7	Cu_2	N_9	103.3(2)	N_8	C_{19}	C_{20}	122.3(6)
N_8	Cu_2	O_6	91.0(2)	C_{20}	C_{19}	C_{18}	122.6(7)
N_8	Cu_2	O_7	92.6(2)	C_{21}	C_{20}	C_{19}	118.1(8)

Table S5 Bond Angles for **2**.

Atom	Atom	Atom	Angle/	Atom	Atom	Atom	Angle/
N_8	Cu_2	N_7	168.3(2)	C_{22}	C_{21}	C_{20}	120.1(8)
N_8	Cu_2	N_9	81.5(2)	C_{21}	C_{22}	C_{23}	119.5(7)
N_9	Cu_2	O_6	91.8(2)	N_8	C_{23}	C_{22}	122.0(8)
O_{20}	Cl_3	O_{21A}	91.2(16)	C_{25}	C_{24}	N_7	110.1(5)
O_{20}	Cl_3	O_{23}	108.7(4)	O_6	C_{25}	C_{24}	106.5(6)
O_{20A}	Cl_3	O_{20}	120(2)	N_7	C_{26}	C_{27}	109.5(6)
O_{20A}	Cl_3	O_{21A}	108(3)	O_7	C_{27}	N_6	119.5(6)
O_{20A}	Cl_3	O_{22}	128(2)	O_7	C_{27}	C_{26}	119.8(6)
O_{21}	Cl_3	O_{20}	112.9(5)	N_6	C_{27}	C_{26}	120.7(7)
O_{21}	Cl_3	O_{23}	103.9(6)	C_{29}	C_{28}	N_6	112.9(7)
O_{22}	Cl_3	O_{20}	111.2(4)	C_{28}	C_{29}	C_{30}	109.3(7)
O_{22}	Cl_3	O_{21}	113.9(6)	N_6	C_{31}	C_{32}	111.5(6)
O_{22}	Cl_3	O_{21A}	78.8(18)	O_9	C_{32}	N_4	122.3(7)
O_{22}	Cl_3	O_{23}	105.6(5)	O_9	C_{32}	C_{31}	122.3(6)
C_1	O_1	Cu_1	104.9(5)	N_4	C_{32}	C_{31}	115.4(7)
C_4	O_2	Cu_1	116.0(4)	N_4	C_{33}	C_{34}	114.4(6)
C_{14}	O_5	C_{13}	117.8(5)	O_{10}	C_{34}	N_5	125.3(8)
C_{25}	O_6	Cu_2	107.6(4)	O_{10}	C_{34}	C_{33}	122.1(7)
C_{27}	O_7	Cu_2	115.0(4)	N_5	C_{34}	C_{33}	112.6(7)
C_{37}	O_8	C_{36}	118.9(7)	N_4	C_{35}	C_{36}	114.2(8)
C_2	N_1	Cu_1	112.1(5)	O_8	C_{36}	C_{35}	107.8(7)
C_2	N_1	C_3	110.7(6)	O_8	C_{37}	C_{38}	125.2(8)
C_3	N_1	Cu_1	110.2(4)	N_2	C_{37}	O_8	111.7(7)

Table S5 Bond Angles for **2**.

**Atom**	**Atom**	**Atom**	**Angle/**	**Atom**	**Atom**	**Atom**	**Angle/**
C_{37}	N_2	Cu_1	130.4(7)	N_2	C_{37}	C_{38}	123.1(10)
C_{37}	N_2	C_{41}	116.8(7)	C_{39}	C_{38}	C_{37}	117.8(9)
C_{41}	N_2	Cu_1	112.8(5)	C_{38}	C_{39}	C_{40}	122.3(9)
C_{42}	N_3	Cu_1	115.4(5)	C_{41}	C_{40}	C_{39}	116.5(10)
C_{46}	N_3	Cu_1	126.3(6)	N_2	C_{41}	C_{40}	123.4(8)
C_{46}	N_3	C_{42}	118.2(7)	N_2	C_{41}	C_{42}	115.2(7)
C_{32}	N_4	C_{33}	123.3(7)	C_{40}	C_{41}	C_{42}	121.4(9)
C_{32}	N_4	C_{35}	119.9(6)	N_3	C_{42}	C_{41}	114.1(8)
C_{35}	N_4	C_{33}	116.8(5)	N_3	C_{42}	C_{43}	121.1(8)
C_{27}	N_6	C_{28}	123.9(6)	C_{43}	C_{42}	C_{41}	124.8(8)
C_{27}	N_6	C_{31}	118.9(6)	C_{42}	C_{43}	C_{44}	120.4(9)
C_{31}	N_6	C_{28}	117.2(6)	C_{45}	C_{44}	C_{43}	118.6(9)
C_{24}	N_7	Cu_2	110.3(4)	C_{44}	C_{45}	C_{46}	118.9(9)
C_{26}	N_7	Cu_2	111.1(4)	N_3	C_{46}	C_{45}	122.6(8)
C_{26}	N_7	C_{24}	111.8(5)	O_{13}	Cl_1	O_{12}	100.5(6)
C_{19}	N_8	Cu_2	114.9(4)	O_{13}	Cl_1	O_{12A}	89.7(7)
C_{19}	N_8	C_{23}	118.1(6)	O_{14}	Cl_1	O_{12}	93.9(6)
C_{23}	N_8	Cu_2	126.4(6)	O_{14}	Cl_1	O_{12A}	79.8(10)
C_{14}	N_9	Cu_2	129.1(5)	O_{14}	Cl_1	O_{13}	114.7(5)
C_{14}	N_9	C_{18}	117.8(6)	O_{14}	Cl_1	O_{15}	122.2(5)
C_{18}	N_9	Cu_2	112.3(4)	O_{15}	Cl_1	O_{12}	98.4(6)
C_9	N_{11}	C_{10}	121.9(7)	O_{15}	Cl_1	O_{12A}	78.2(10)
C_9	N_{11}	C_{12}	120.3(6)	O_{15}	Cl_1	O_{13}	117.9(4)

Table S5 Bond Angles for **2**.

**Atom**	**Atom**	**Atom**	**Angle/**	**Atom**	**Atom**	**Atom**	**Angle/**
C12	N11	C10	117.6(5)	O16A	Cl2	O17	118.1(19)
C4	N12	C5	125.7(6)	O16A	Cl2	O18	123.5(19)
C4	N12	C8	117.2(6)	O16A	Cl2	O19A	106(2)
C5	N12	C8	117.1(6)	O17	Cl2	O16	108.6(7)
O1	C1	C2	108.0(7)	O17	Cl2	O19A	105.5(13)
N1	C2	C1	110.9(6)	O18	Cl2	O16	105.5(8)
C4	C3	N1	108.1(6)	O18	Cl2	O17	110.2(4)
O2	C4	N12	120.9(6)	O18	Cl2	O19	115.6(9)
O2	C4	C3	120.3(6)	O18	Cl2	O19A	86.9(17)
N12	C4	C3	118.9(7)	O19	Cl2	O16	108.3(7)
N12	C5	C6	121.4(10)	O19	Cl2	O17	108.4(5)
N12	C5	C6A	102.8(9)	O24	Cl4	O26	102.8(6)
C5	C6	C7	105.8(10)	O24	Cl4	O27	115.6(4)
C7A	C6A	C5	115.9(14)	O25	Cl4	O24	116.9(5)
N12	C8	C9	110.9(6)	O25	Cl4	O26	97.8(6)
O3	C9	N11	121.5(7)	O25	Cl4	O27	117.4(6)
O3	C9	C8	122.8(6)	O27	Cl4	O26	101.7(6)
N11	C9	C8	115.7(6)	N13	C47	C48	162(7)
N11	C10	C11	113.6(6)	N13	C47A	C48A	169(6)
O4	C11	N10	124.3(8)				
